# Dataset of the net primary production on the Qinghai-Tibetan Plateau using a soil water content improved Biome-BGC model

**DOI:** 10.1016/j.dib.2019.104740

**Published:** 2019-11-04

**Authors:** Chuanhua Li, Hao Sun, Xiaodong Wu, Haiyan Han

**Affiliations:** a College of Geography and Environmental Science, Northwest Normal University, Lanzhou, 730070, China; b Cryosphere Research Station on the Qinghai-Tibetan Plateau, State Key Laboratory of Cryospheric Science, Northwest Institute of Eco-Environment and Resources, Chinese Academy of Sciences, Lanzhou, 730000, China; c University of Chinese Academy Sciences, 19(A) Yuquan Road, Shijingshan District, Beijing, 100049, China

**Keywords:** Soil water, Biome-BGC, Net primary production, Freeze-thaw process, Qinghai-Tibetan Plateau

## Abstract

The Biome-BGC (biome biogeochemical cycles) model is widely used for modeling the net primary productivity (NPP) of ecosystems. However, this model ignores soil water changes during the freeze-thaw process in permafrost regions, which may lead to considerable errors in the NPP estimations. In this context we propose a numerical simulation method for improving soil water content during the freeze-thaw process based on the field observation data of soil water and temperature. This approach does not require new parameters and has no impact on other modules. The improvement of soil water content during the freeze-thaw process was then incorporated in the Biome-BGC model for NPP in an alpine meadow in the central Qinghai-Tibetan Plateau (QTP). Interpretation of this data can be found in a research article entitled “An approach for improving soil water content for modeling net primary production on the Qinghai-Tibetan Plateau using Biome-BGC model” (Li et al., 2019).

**Table:** Specifications Table

Subject	Ecological Modelling
Specific subject area	Net Primary Productivity, Permafrost Region
Type of data	Table
How data were acquired	Model results Analysis data
Data format	Raw and Analysed
Parameters for data collection	The Tanggula station is located in the middle of Qinghai-Tibetan Plateau, and its climate condition is close to the multi-year average level of Qinghai-Tibetan Plateau. The main vegetation is alpine meadow, which is the constructive species of Qinghai Tibet Plateau. It provides a good opportunity to study the applicability of Biome-BGC model in the Qinghai- Tibetan Plateau.
Description of data collection	In this dataset, there are four files, 1) the average sunshine shortwave radiant flux density and sunshine hours were calculated by the MTCLIM4.0 model on the Tanggula site, which located in the central Qinghai-Tibetan Plateau; 2) the outputs from the original model; 3) the outputs from the improved model, and 4) the analysis and validation data.
Data source location	Cryosphere Research Station on the Qinghai-Tibetan Plateau, State Key Laboratory of Cryospheric Science, Northwest Institute of Eco-Environment and Resources, Chinese Academy of Sciences Qinghai-Tibetan Plateau, China 91°54′E, 32°51′N
Data accessibility	Repository name: Mendeley Data DOI: 10.17632/jky2frt4pk.4 Direct URL to data: https://data.mendeley.com/datasets/jky2frt4pk/4
Related research article	Chuanhua Li, Hao Sun, Xiaodong Wu, Haiyan Han. Approach for improving soil water content for modeling net primary production on Qinghai-Tibetan Plateau using Biome-BGC model [1]. DOI:https://doi.org/10.1016/j.catena.2019.104253

**Table:** 

**Data Value** • The Qinghai-Tibetan Plateau is the largest permafrost region in the high altitude area in the world. The data include information about net primary productivity in the permafrost region of Qinghai-Tibetan Plateau from 2012 to 2014. • The data contain all the driving data and outputs of the Biome-BGC model, which are helpful to analyze the structure and pattern of soil moisture in permafrost area. • These data may be helpful for comparative analysis in other permafrost areas. • These data can improve our understanding of vegetation growth in permafrost region under the background of climate warming.

## 1 Data

The “MTCLIM-XL_0.4 Tanggula.xlsx” includes the average solar shortwave radiation flux density (SRFD) and daylength (Daylen) calculated from MTCLIM 4.0 model in 2012–2014, which are used to drive the Biome-BGC model. The “Documentation” sheet introduces the model, “Improved. annout” and “Original. annout” are the outputs of each year from the improved model and the original model, and “Improved. dayout” and “Original. dayout” are the results of each day output from the improved model and the original model. “miss5093.ini” sheet is the number of sites, “miss5093.restart” sheet is the parameter obtained after the initial simulation, “miss5093.met” sheet is the daily input meteorological data, “co2” sheet is the concentration of carbon dioxide, “epc” sheet is the physiological and ecological parameters of vegetation. The “analysis data. xlsx” includes soil water data, water stress coefficient data and net primary productivity data.

## 2 Experimental design, materials, and methods

The Biome-BGC was created by the Numerical Terradynamic Simulation Group (NTSG), University of Montana. It is a biogeochemical model that simulates the storage and flux of water, carbon, and nitrogen between the ecosystem and the atmosphere, and within the components of the terrestrial ecosystem. This model also can simulate photosynthesis, respiration, allocation of organic matter, litter and decomposition of plant tissues, and circulation and migration of nutrients in different ecosystems [2].

The Biome-BGC is driven by three types of parameters: site parameters (Table 1), daily meteorological data, and plant physiology and ecological parameters. Soil texture, latitude, longitude, and altitude, were obtained from field measurements. The atmospheric carbon dioxide concentration data were based on relevant literatures [3,4].

**Table 1:** Site parameters for Biome-BGC model.

name	value	type	Description
**SIM_CONTROL**			
EPC_COLUMN	c3grass	String	Ecophysiological constants column in the ‘epc’ worksheet that describes the vegetation
SIM_YEARS	11	Integer	Number of years to simulate
FIRST_SIM_YEAR	2005	Integer	First year of simulation
PROGRESS	TRUE	Boolean	Display progress information?
SIM_TYPE	2	Integer	Simulation Type: 1 = Spinup, 0 = Normal, 2 = Spin and Go
**CLIM_CHANGE**			
TMAX_OFFSET	0	Float	Additional offset for maximum temperature
TMIN_OFFSET	0	Float	Additional offset for minimum temperature
PRCP_MULT	1	Float	Multiplier for precipitation (dim)
VPD_MULT	1	Float	Multiplier for VPD (dim)
**CO2_CONTROL**			
CO_2__FLAG	1	Boolean	0 = Use a fixed CO_2_ concentration, 1 = Use CO_2_ values in the CO_2__WORKSHEET
**SITE**			
SOIL_DEPTH	0.6	Float	Soil Depth (meters)
SOIL_SAND	67	Float	Soil Sand (%)
SOIL_SILT	30	Float	Soil Silt (%)
SOIL_CLAY	3	Float	Soil Clay (%)
ELEV	5100	Integer	Elevation (meters)
LAT	32.9	Float	Latitude (decimal degrees)
LON	91.9	Float	Longitude (decimal degrees)
**SITE_N**			
RAMP_NDEP_FLAG	0	Boolean	Do nitrogen deposition ramping?
REF_NDEP_YEAR	2000	Integer	Reference year for nitrogen deposition
IND_NDEP	0.0001	Float	Industrial nitrogen deposition
NDEP	0.0001	Float	Pre-industrial nitrogen deposition
NFIX	0.0006	Float	Nitrogen fixation
**SITE_W**			
INIT_SNOWW	10	Float	Initial snow wate
INIT_SOILW	0.5	Float	Initial soil water as a fraction of saturation
**SITE_C**			
FY_MAX_LEAFC	0.001	Float	First year maximum leaf carbon
FY_MAX_STEMC	0	Float	First year maximum stem carbon
**OUTPUT**			
DO_DAILY	TRUE	Boolean	Do daily outputs?
DO_ANNUAL	TRUE	Boolean	Do annual summary outputs?
NUM_VARS	9	Integer	Number of output variables beginning on the next row
1	GPP	String	Gross Primary Productivity
2	NPP	String	Net Primary Productivity
3	MR	String	Net Ecosystem Exchange
4	ET	String	Evapotranspiration
5	OF	String	Soil water outflow
6	PRCP	String	Precipitation
7	LAI	String	Leaf Area Index
8	TSOIL	String	Soil temperature
9	SOILW	String	Soil water

The plant physiology and ecological parameters include both labeled and unlabeled parameters (Table 2). The labeled parameters are Boolean and do not require sensitivity analysis. The unlabeled parameters were analyzed for sensitivity. In this study, the sensitivity parameters of the Biome-BGC model were analyzed based on the coefficient of variation, and the parameters with coefficients of variation greater than 0.1 were field measurements. The SLA (specific leaf area) was calculated by scanning the field samples. Specifically, the leaf samples were collected at each sampling site, and then the samples were scanned by a scanner to obtain the digital images. The area of each leaf was measured, and then the sample was dried to measure the dry weight. The ratio of average leaf area to dry weight was the specific leaf area. The contents of carbon and nitrogen in the sample were analyzed using an automatic chemical analyzer (SmartChem 2000, Alliance, France).

**Table 2:** Plant physiology and ecological parameters.

Keyword	c3grass	Type	Description
WOODY_FLAG	0	(flag)	1 = WOODY 0 = NON-WOODY
EVERGRN_FLAG	0	(flag)	1 = EVERGREEN 0 = DECIDUOUS
C3_FLAG	1	(flag)	1 = C3 PSN 0 = C4 PSN
MODEL_PHEN_FLAG	0	(flag)	1 = MODEL PHENOLOGY 0 = USER-SPECIFIED PHENOLOGY
ONDAY	120	*(yday)	yearday to start new growth
OFFDAY	290	*(yday)	yearday to end litterfall
TRNS_GR_PROP	1	*(prop.)	transfer growth period as fraction of growing
LIT_FALL_PROP	1	*(prop.)	litterfall as fraction of growing season
LFR_TURNOVER	1	(1/yr)	annual leaf and fine root turnover fraction
LWOOD_TURNOVER	0	(1/yr)	annual live wood turnover fraction
MORT_FRAC	0.1	(1/yr)	annual whole-plant mortality fraction
FIRE_MORT_FRAC	0.001	(1/yr)	annual fire mortality fraction
ALLOC_FR_LEAF	0.881	(ratio)	new fine root C: new leaf C
ALLOC_STEM_LEAF	0	(ratio)	new stem C: new leaf C
ALLOC_LWOOD_TOTWOOD	0	(ratio)	new live wood C: new total wood C
ALLOC_CROOT_STEM	1.0263	(ratio)	new croot C: new stem C
GR_PROP	0.5	(prop.)	current growth proportion
LEAF_CN	33.7356	(kgC/kgN)	C:N of leaves
LLITTER_CN	49.7	(kgC/kgN)	C:N of leaf litter, after retranslocation
FR_CN	49.7	(kgC/kgN)	C:N of fine roots
LWOOD_CN	0	(kgC/kgN)	C:N of live wood
DWOOD_CN	0	(kgC/kgN)	C:N of dead wood
LIT_LAB_PROP	0.39	(DIM)	leaf litter labile proportion
LIT_CEL_PROP	0.44	(DIM)	leaf litter cellulose proportion
LIT_LIG_PROP	0.17	(DIM)	leaf litter lignin proportion
FR_LAB_PROP	0.3	(DIM)	fine root labile proportion
FR_CEL_PROP	0.45	(DIM)	fine root cellulose proportion
FR_LIG_PROP	0.25	(DIM)	fine root lignin proportion
DWOOD_CEL_PROP	0.76	(DIM)	dead wood cellulose proportion
DWOOD_LIG_PROP	0.24	(DIM)	dead wood lignin proportion
CANOPYW_INT_COEF	0.021	(1/LAI/d)	canopy water interception coefficient
CANOPY_LT_EXT_COEF	0.48	(DIM)	canopy light extinction coefficient
LEAF_AREA_RAT	2	(DIM)	all-sided to projected leaf area ratio
AVG_SLA	12.35	(m2/kgC)	canopy average specific leaf area
SHADE_SUN_SLA_RAT	2	(DIM)	ratio of shaded SLA:sunlit SLA
FLNR	0.21	(DIM)	fraction of leaf N in Rubisco
GS_MAX	0.006	(m/s)	maximum stomatal conductance
GC_MAX	0.00001	(m/s)	cuticular conductance (projected area basis)
GB	0.04	(m/s)	boundary layer conductance (projected area basis
PSI_MIN	−0.6	(MPa)	leaf water potential: start of conductance reduction
PSI_MAX	−2.3	(MPa)	leaf water potential: complete conductance reduction
VPD_MIN	930	(Pa)	vapor pressure deficit: start of conductance reduction
VPD_MAX	4100	(Pa)	vapor pressure deficit: complete conductance reduction

The description of soil water cycle in the original model involves several processes, such as canopy interception and evaporation, soil water potential energy and content, stomatal conductance and evapotranspiration, surface evaporation, flooding, and infiltration. The calculation of soil water content in the original model can be summarized as follows:

(1) $$W_{vwc}=f_{vwc}\left(P,k_{int},L_{A},E_{int},t,VPD,\psi ,\theta ,PPFD\ldots \;\right)\times 1000\times d_{soil}$$

where $f_{vwc}\left(P,k_{int},L_{A},E_{int},t,VPD,\psi ,\theta ,PPFD\ldots \;\right)\;$ is the soil volumetric water content, *P* is the precipitation, *k*_int_ is the canopy interception coefficient, *L*_A_ is the leaf area index, *E*_int_ is the canopy interception evaporation coefficient, *t* is the temperature, *VPD* is the water pressure, ψ is the soil water potential energy, θ is the field water holding capacity, *PPFD* is the stomatal conductance, and $d_{\text{soil}}$ is the soil depth. The improved formula is as follows:

(2) $$W_{vwc}=f_{vwc}\left(P,k_{int},L_{A},E_{int},t,VPD,\psi ,\theta ,PPFD\ldots \;\right)\times 1000\times d_{soil}\;+f_{FTW}\left(T_{soil}\right)$$

where $f_{FTW}\left(T_{soil}\right)$ is the freeze-thaw water content and $T_{\text{soil}}$ is the soil temperature.
